# MCN-CPI: Multiscale Convolutional Network for Compound–Protein Interaction Prediction

**DOI:** 10.3390/biom11081119

**Published:** 2021-07-29

**Authors:** Shuang Wang, Mingjian Jiang, Shugang Zhang, Xiaofeng Wang, Qing Yuan, Zhiqiang Wei, Zhen Li

**Affiliations:** 1 College of Computer Science and Technology, China University of Petroleum, Qingdao 266580, China; 20210006@upc.edu.cn; 2 School of Information and Control Engineering, Qingdao University of Technology, Qingdao 266520, China; jiangmingjian@qut.edu.cn; 3 College of Computer Science and Technology, Ocean University of China, Qingdao 266100, China; zsg@ouc.edu.cn (S.Z.); 21170231282@stu.ouc.edu.cn (X.W.); yuanqing@stu.ouc.edu.cn (Q.Y.); weizhiqiang@ouc.edu.cn (Z.W.); 4 College of Computer Science and Technology, Qingdao University, Qingdao 266071, China

**Keywords:** compound–protein interaction, drug screening, convolutional network, deep learning

## Abstract

In the process of drug discovery, identifying the interaction between the protein and the novel compound plays an important role. With the development of technology, deep learning methods have shown excellent performance in various situations. However, the compound–protein interaction is complicated and the features extracted by most deep models are not comprehensive, which limits the performance to a certain extent. In this paper, we proposed a multiscale convolutional network that extracted the local and global features of the protein and the topological feature of the compound using different types of convolutional networks. The results showed that our model obtained the best performance compared with the existing deep learning methods.

## 1. Introduction

Drug discovery contains multiple steps that take a long time and cost a lot of money. Predicting and identifying the compound–protein interaction (CPI) play an essential role in the discovery and development of safe and effective new drugs. In the initial stage of drug discovery, screening out the compounds that interact with the target protein can greatly increase the success rate of drug discovery [1]. A large number of studies have shown that the advantage of deep learning is that it can obtain a robust descriptor of the original data after nonlinear transformation [2], which could promote the model to learn the task-related features from the data. With the establishment of more and more datasets of protein structures and compound–protein interactions, more and more studies have attempted to introduce deep learning methods into both drug discovery [3,4,5] and the predictive task of compound–protein interaction [6,7,8].

These methods usually integrated the information of proteins and molecules into one framework for binary classification. The existing deep learning methods for compound–protein interaction can be summarized into three categories:

1. The predictive models based on a 1D structure.

The protein is composed of multiple amino acids, and each amino acid is represented by one character or multiple characters. A protein could be expressed as a string of multiple characters. The 1D sequence of the protein is similar, with the text in the field of natural language processing. Therefore, some researchers tried to apply the end-to-end representation learning method in order to learn the feature of protein sequences.

DeepDTA [9] built a model to predict the binding affinity between ligands and proteins, in which the protein was represented as a 1D amino acid sequence. The results showed that the performance of the model based on deep learning was superior to the models based on traditional machine learning. A MFDR model [10] used a multi-scale protein sequence descriptor to extract the feature of the 1D amino acid sequence, which was combined with the feature of the molecular fingerprint in order to predict the interaction of the compound–protein. Tsubaki et al. [11] applied 1DCNNs and GCN to learn features from the 1D amino acid and molecular graph, respectively, for predicting compound–protein interactions. The CGBVS-DNN [12] and DBN [13] model also extracted the feature of the protein from the 1D amino acid sequence.

2. The predictive models based on a 2D structure.

Recently, some studies constructed a 2D structure descriptor through a protein sequence and extracted features based on it or based on an original secondary structure in various tasks. Fout et al. [14] introduced the protein graph into the prediction of the protein–protein interaction, in which the basic node in the graph corresponded to the residue. DrugVQA [15] was a compound–protein interaction predictive model in which the protein was described as a distance matrix through the amino acid sequence. DGraph-DTA [16] built a contact map to represent the protein based on the protein sequence in order to predict the binding affinity. Instead of considering the residue as the node, ProteinGCN [17] built a protein graph according to the distance between atoms and regarded the atom as the basic node. iProStruct2D [18] performed protein classification based on 2D snapshots taken from the 3D structure. SSnet [19] extracted the feature from the secondary structure of the protein in order to predict the compound–protein interaction, which was based on the original 2D information of the protein.

3. The predictive models based on a 3D structure.

The structure of an active protein is not a simple combination of amino acids, but a 3D structure formed by protein folding. The stability of the 3D structure benefits from the interaction between amino acids, which also affects the compound–protein interaction. Researchers tried to learn the interaction from the 3D structure of the compound–protein complex. AtomNet [20] was the first model that utilized deep learning methods to predict the binding affinity of molecules and proteins based on 3D structural information. In the AtomNet, the author extracted the feature from the 3D grids of the compound–protein complex. The 3DCNN [21] and Se-OnionNet [22] also predicted the binding affinity of the protein and the molecule based on the complex, which was obtained from the docking software. ACNN [23] constructed a neighbor distance matrix using atomic coordinates and a distance based on the complex in order to predict the free energy. However, there are only 17679 biomolecular complexes of the protein–ligand [24]. Due to the fact that there is a certain deviation in the calculation of the docking software, it is not accurate enough to predict interactions based on the complex conformation obtained from this software. Although researchers predicted the interaction between the proteins and the compounds through the 3D structure, the accuracy needs to be improved.

The spatial structure feature of the binding site provides precise information for the binding between proteins and compounds, but the global feature of the protein may also affect the binding process. It is mainly embodied in two aspects. First, in consideration of the shape and volume of the protein, the binding site only takes up a small spatial proportion of the protein volume. The choice of the compound is influenced by the global feature of the protein. Second, in the process of binding, the folding mode of protein conformation is more complicated. The formation of the binding site is the result of the combined effect of many factors. Atoms that do not belong to the binding site may also affect binding.

Based on the consideration of the above factors, this work not only considers the local feature of the binding site that closely relates to the docking process, but also considers the global feature of the protein. Due to the large volume of the protein and the complex spatial structure that could result in high computational complexity, the 3D spatial structure of the whole protein is discarded. The 1D amino acid sequence is used to represent the global feature. In this work, the local feature from the binding site and the global feature from the amino acid sequence are processed by two convolutional neural networks to extract the information of the protein. Besides, the graph convolutional network is used to extract the feature of the compound. All these features are combined to predict the compound–protein interaction.

## 2. Materials and Methods

**Figure 1:**
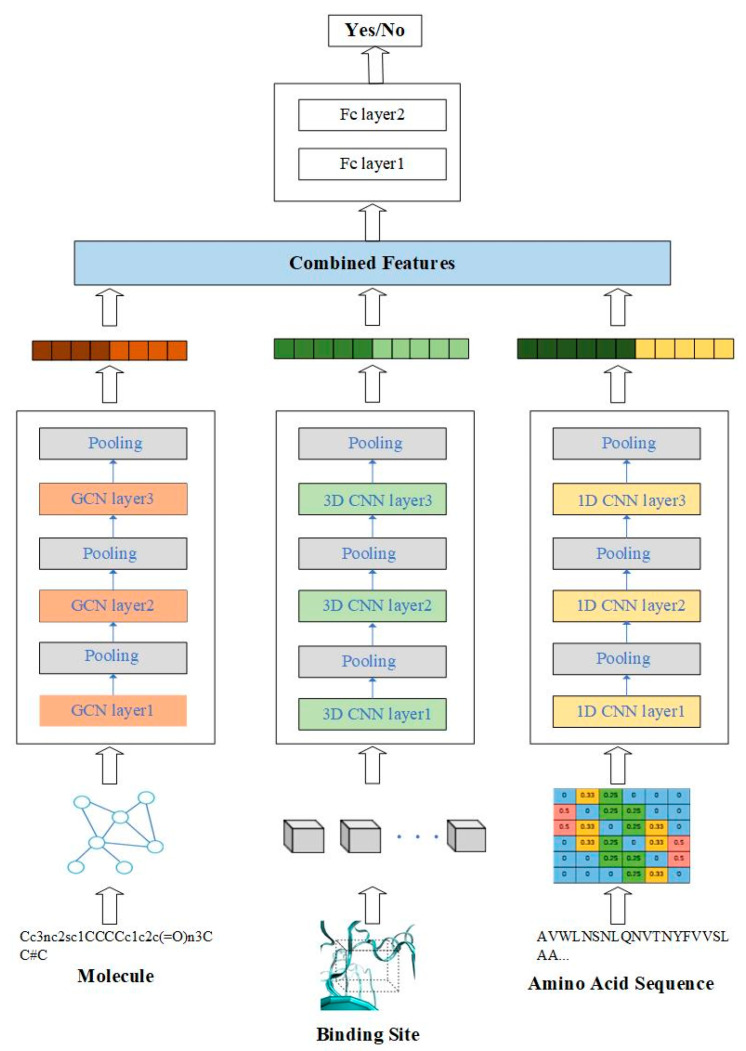
Overview of the multiscale convolutional network (MCN) for compound–protein interaction prediction.

In this work, we propose a predictive model for compound–protein interaction using three convolutional neural networks. The architecture is shown in Figure 1. In this architecture, the features of compound–protein interaction contain three parts, including the local feature of the binding site, the global feature of the 1D amino acid sequence and the topological feature of the molecule. The local feature of the protein indicates the spatial feature of the binding site through 3D convolutional neural network (3D-CNN), which could discover the precise binding information. The global feature of the protein indicates the feature from amino acid sequence using 1D convolutional neural network. Moreover, in order to discover topological structure information of the molecule, the graph convolutional network is applied to extract the relationship between atoms. All three features are combined to predict compound–protein interaction.

### 2.1. The Local Feature of the Protein Based on Multi-Channel 3D Convolutional Neural Network

The effective binding of the compound and the protein is the key to the function of the drug. The binding site plays an important role in the process of binding. The essence of the combination of the protein and molecule is that the atoms of the molecule interact with the atoms of the binding site. The focus of this work is finding the molecules that interact with the binding site of the protein. Therefore, in the description of the protein, the main information is the characteristics of the binding site. Since the formation of the binding site is based on its 3D structure, we extract features from the 3D structure of the binding site.

For the spatial structure, the first problem that needs to be solved is how to construct the spatial descriptor of the binding site in order to extract effective information. In the 3D spatial structure, angstrom (Å) is used as the basic unit of the measurement. Inspired by image pixels in the field of image processing, the protein is represented by the basic units of each voxel with a size of 1 Å × 1 Å × 1 Å. The method of using voxels to represent proteins has been used in the docking scoring AutoDock Vina [25] in the virtual screening and DeepSite [26] model for predicting the position of the binding site.

In the compound–protein complex composed of the protein and the molecule, non-covalent bond interactions play an important role in the formation of the protein conformation, the stability of the protein conformation and the stability and activity of the binding of the protein and the molecule. Similar to DeepSite [26], the features of eight channels related to these non-covalent bond interactions are extracted to act as an important part of the protein descriptor, including hydrophobic, aromatic, hydrogen bond acceptor, hydrogen bond donor, positive ionizable, negative ionizable, metal and excluded volume.

The occupancy of a single atom is calculated as follows:

(1) $$n\left(r\right)=1-\exp\left(-{\left(\frac{r_{vdw}}{r}\right)}^{12}\right)$$

where $r_{vdw}$ is the Van der Waals atom radius and r is the distance.

The calculation method of the above descriptors of the protein has been integrated into HTMDs [27]. In DeepSite [26], the values of eight channels are calculated for all atoms of the protein in order to predict the location of the binding site. However, the interaction between the protein and the molecule mainly occurs in the binding site. We focused on studying the role of binding sites and molecules. Therefore, in the 3D descriptors of the protein, the voxel values of the eight channels are only calculated for the atoms belonging to the binding site.

**Figure 2:**
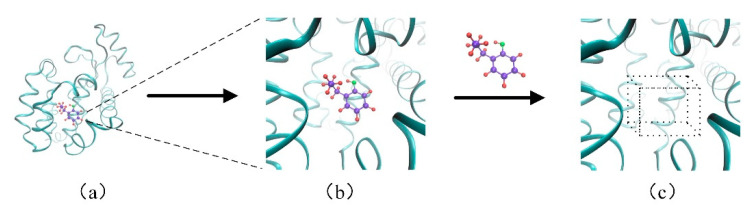
The descriptor of the binding site. (**a**)The binding site occupies a small space in the complex of the protein and the molecule; (**b**) The binding site and the molecule are connected by non-covalent bond; (**c**) The constructed box of the binding site.

The binding site occupies a small space in the whole protein, shown in Figure 2a. The binding site and the molecule are connected by non-covalent bond, and the rest of the proteins have little contribution on this connection, which is shown in Figure 2b. If we build an eight-channel grid for all atoms in the protein, it could cause a waste of computing resources. Therefore, only the information of the binding site was extracted and the corresponding descriptors were constructed. In order to facilitate the feature extraction of the binding site, a box (30 Å × 30 Å × 30 Å) was constructed around the binding site, which is shown in Figure 2c. According to eight channels of the protein in the previous section, the feature of the binding site was extracted from the eight channels to cover different properties.

**Figure 3:**
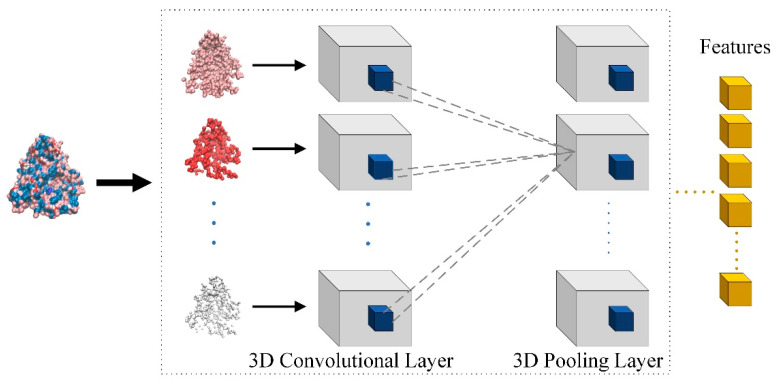
Feature extraction of the binding site based on multi-channel 3D convolutional neural network.

Through the descriptor of the binding site, the atoms belonging to the binding site were divided into eight different channels and many voxels were constructed with DeepSite. Thus, the descriptor of the binding site is represented by the cube box composed of voxels of eight channels. Furthermore, the model uses 3D convolutional neural networks to process these cube boxes to obtain the feature of the binding site, which is shown in Figure 3. If the size of the 3D convolutional kernel is (*P*, *Q*, *R*), the output at the position (*x*, *y*, *z*) of the feature cube is calculated as the following equation.

(2) $$v_{\left(l+1\right)}^{xyz}=\underset{m}{\sum }\overset{P-1}{\underset{i=0}{\sum }}\overset{Q-1}{\underset{j=0}{\sum }}\overset{R-1}{\underset{k=0}{\sum }}w_{l}^{ijk}v_{l}^{\left(x+p\right)\left(y+q\right)\left(z+r\right)}+b_{l}$$

where $w_{l}^{ijk}$ represents the weight of the position (i,j,k) in the l-th layer 3D convolutional kernel, $v_{l}^{\left(x+p\right)\left(y+q\right)\left(z+r\right)}$ represents the feature values in the l-th layer at the feature cube position (x+p)(y+q)(z+r), $b_{l}$ represents the bias of the l-th layer and $v_{\left(l+1\right)}^{xyz}$ represents the value of (*x*, *y*, *z*) in the (l+1)-th layer 3D feature cube. The bias and weight of each layer are obtained through training. The maximum pooling is utilized in the proposed model.

### 2.2. The Global Feature of the Protein Based on 1D Convolutional Neural Network

The protein sequence is a representation of the primary structure of a protein, which consists of multiple amino acids. There are 20 kinds of known amino acids, and each amino acid is usually represented by a three-letter string or one character. Since proteins are a biomacromolecule, there are many amino acids in a single protein. The amino acid sequence expressed in characters is similar to the text. In our work, each protein is represented by 1000 characters in length. If the number of amino is less than 1000, the sequence will be filled with 0, and if the number is more than 1000, the sequence will be cut. The amino acid sequence is vectorized in a manner similar to text processing. The word embedding is used to convert each amino acid into a 128-dimensional vector. Thus, the amino acid sequence is transformed into a feature matrix with 128 × 1000 size.

**Figure 4:**
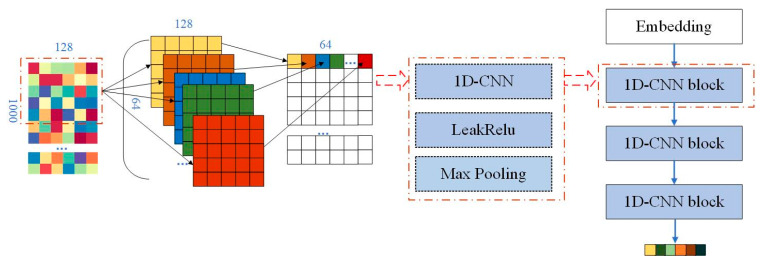
The global feature extraction from the protein based on 1D convolutional neural network.

The 1D convolutional neural networks, which are composed of three 1D convolutional blocks, are utilized to extract the global feature. Each convolutional block includes one convolutional layer, one LeakyRelu function and one maximum pooling layer. The convolutional process is shown in Figure 4. Take the first convolutional layer as an example. The size of the convolutional kernel is 5, and 64 convolutional kernels are utilized in the first convolutional layer.

The convolutional process for the amino acid sequences is as follows:

(3) $$s_{\left(l+1\right)}^{k}=\overset{N-1}{\underset{j=0}{\sum }}s_{l}^{\left(k-j\right)}w_{l}^{j}+b_{l}$$

where $s_{l}^{\left(k-j\right)}$ is the feature vector of amino acid sequence at the position (k−j)  in the  l-th layer, $w_{l}^{j}$ is the corresponding convolutional kernel and $b_{l}$ is the bias.

### 2.3. The Molecular Feature Based on Graph Convolutional Network

The molecule is described as a graph with topological connection. Each node in the graph corresponds to an atom, and the edge corresponds to a chemical bond between atoms in the molecule. The atom has many attributes, such as atom type, atomic degree, number of connected hydrogen atoms, etc. These attributes, which are regarded as the feature of atoms, are described as one-hot vectors. The details are shown in Table 1.

One molecule is described as G=(V,E), where V corresponds to all atoms in the molecule and E is the set of bonds. For the atomic node i, its feature is represented as $x_{i}$. The feature of the molecular graph is expressed as $X_{N\times L}$, where N represents the number of nodes and L represents the feature dimension of each node. The topological connection of molecules is represented by the adjacency matrix $A_{N\times N}$. If there is a connection between the node i and the node j, then $A_{\left(i,j\right)}=1$; otherwise $A_{\left(i,j\right)}=0$. In addition, the number of adjacent atoms connected to each atom in the molecular graph is recorded in the degree matrix.

A graph convolutional network [28] is performed on the molecular graph in order to extract the molecular feature. The convolutional method is composed of three convolutional layers and three pooling layers. The convolutional operation in each layer is calculated as follows:

(4) $$H_{\left(l+1\right)}=\sigma \left({\tilde{D}}^{-\frac{1}{2}}\tilde{A}{\tilde{D}}^{-\frac{1}{2}}H_{l}W_{l}\right)$$

where $\tilde{A}=A+I_{N}\;$ refers to the adjacency matrix of undirected molecular graphs containing self-connection, D is the degree matrix of the molecular graph, ${\tilde{D}}_{ii}=\underset{j}{\sum }{\tilde{A}}_{ij}.W_{l}\;$ is the weight matrix and σ(.) is the activative function.

## 3. Model Training

**Figure 5:**
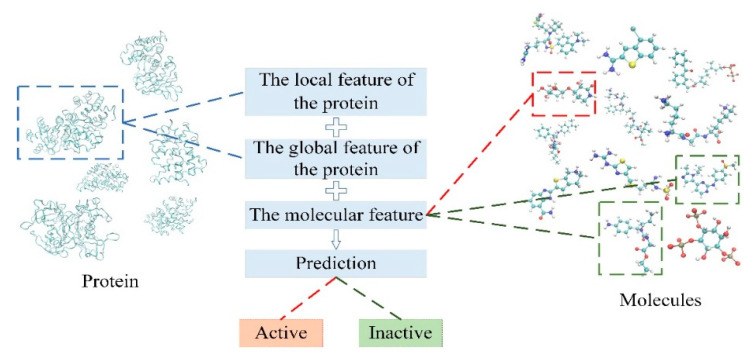
The prediction of compound–protein interaction.

In the predictive task of the compound–protein interaction, the local feature of the protein from the binding site is obtained through the multi-channel 3D convolutional neural network. The global feature of the protein from the amino acid sequence is obtained through the 1D convolutional neural network, and the molecular feature is obtained through the graph convolutional network. These three features are combined as Equation (5) to predict the compound–protein interaction in order to determine whether the molecule can effectively dock with the protein. The overall process is shown in Figure 5.

(5) $$X_{CPI}=\left[X_{L},X_{G},X_{M}\right]$$

where $X_{L}$ indicates the local feature of the protein, $X_{G}$ indicates the global feature of the protein and $X_{M}$ indicates the molecular feature.

Molecules that can bind to the protein are labeled as positive samples, and others are labeled as negative samples. There are hundreds of millions of molecules in nature or generated with the aid of computer-aided technology, but there are a small number of molecules that can effectively bind to specific proteins. A huge imbalance between positive samples and negative samples results in the inefficiency of the training process. In order to minimize the interference caused by the imbalance, the focal loss [29] is introduced as the loss function, which was originally proposed to address the problem of the imbalance in the field of target detection. The focal loss is described as Equation (6).

(6) $$L_{F}=\{\begin{array}{c} -\alpha {\left(1-y^{\prime }\right)}^{\gamma }logy^{\prime },\;\;\;\;\;\;\;\;\;\;\;\;\;\;\;\;\;\;\;\;\;\;y=1\\ -\left(1-\alpha \right)y^{'}{}^{\gamma }log\left(1-y^{\prime }\right),\;\;\;\;\;\;\;\;\;\;\;y=0 \end{array}$$

where α is the balance factor to adjust the proportion of positive and negative samples, γ factor is set to ensure the model pays more attention to samples that are difficult to distinguish and $y^{\prime }$ is the predicted value.

Assume that the number of positive samples is P and the number of negative samples is N. The coefficient of positive samples is $\frac{P}{P+N}$ and the coefficient of negative samples is $\frac{N}{P+N}$. The final loss is calculated as the following equation.

(7) $$L_{m}=\{\begin{array}{c} -\frac{N}{P+N}{\left(1-y^{\prime }\right)}^{\gamma }logy^{\prime },\;\;\;\;\;\;\;\;\;y=1\\ -\frac{P}{P+N}{y^{\prime }}^{\gamma }log\left(1-y^{\prime }\right),\;\;\;\;\;\;\;\;\;\;\;y=0 \end{array}$$

## 4. Results

### 4.1. Dataset

Directory of Useful Decoys, Enhanced (DUD-E) is a dataset that provides 102 unique proteins that correspond to 124 docking molecules on average. As for each docking molecule, 50 decoys are prepared. These decoys own similar properties to the actives. In our experiment, 91 target proteins and their corresponding compounds constitute a dataset, since it is hard to extract the precise channel information from the rest targets. For each target protein, there are multiple docking molecules that are recorded as positive samples, and others that are labeled as negative samples. In the experiment, one sample pair contains one protein, one compound and one label. To compare our model with other models more objectively, the splitting of the dataset followed the same experimental setting of Lim et al. [30] and Tsubaki et al. [11]. The training set and the test set are divided according to the type of protein. For each protein target, the ratio of ligands (positive) and decoys (negative) is set to 1:1. The details are shown in Table 2 and Table 3. There are 29,030 sample pairs in the training dataset and 10,746 sample pairs in the test dataset in total, which contain both positive and negative ones.

### 4.2. The Performance of the Model

In the DUD-E dataset, each sample pair contains a protein, a molecule and a label. The label shows whether the protein and molecule in the sample pair could be docked. If they are docking, the corresponding label is 1; otherwise it is 0. For the test set, given a protein and a molecule, the model needs to predict the docking possibility, which is a classification task. The general ROC AUC is used as the measurement indicator. A higher AUC value indicates a better performance.

In order to evaluate the performance of the proposed model objectively, we compared it with seven other models. These models contain open source molecular docking programs that are widely used in virtual screening tasks (AutoDock Vina [25] and Smina [31]), deep learning models (Tsubaki’s model [11], AtomNet [20], 3D-CNN [21]) and the latest graph-based model (L+LP+R [32] and Lim’s model [30]).

Tsubaki’s model [11] applied 1DCNNs to extract protein features and used GCN to extract molecular features. The AtomNet [20] used a 3D convolutional neural network to extract the combined feature from the 3D grid of the compound–protein complex for the interaction prediction. Similar with AtomNet, the 3D-CNN [21] method also extracted the feature of the complex. The docking poses were obtained from the docking software. The L+LP+R model [32] constructed two topological graphs L and LP based on the binding structure of the protein and molecules. Besides, the author merged the ranking R of the docking posture into the model. Lim’s model [30] embedded structural information of the binding pose in a graph and introduced an attention mechanism into the prediction.

Table 4 shows the comparison of the performance of multiple models on the DUD-E dataset for distinguishing actives and decoys. The AUC values of other models in Table 4 are derived from AtomNet [20], 3D-CNN [21], L+LP+R [32], Tsubaki’s model [11] and Lim’s model [30]. As shown in Table 4, the proposed model obtains the best result. The AUC value (0.975) of our model is higher than the deep learning models, such as AtomNet (0.895), 3D-CNN (0.868), L+LP+R (0.93) and Lim’s model (0.968). Different to the above four models, in the proposed model, the docking structure of the protein and the molecule is not extracted. The features of the protein and the molecule are extracted separately. Generally speaking, the feature extracted by the docking structure of compound–protein complexes could contain more precise information. However, the experimental results indicate that the proposed model is superior to the model that extracts features from the docked complex structure, which further shows that the proposed model is capable of predicting the compound–protein interaction.

### 4.3. The Performance of the Model on Different Proteins

**Figure 6:**
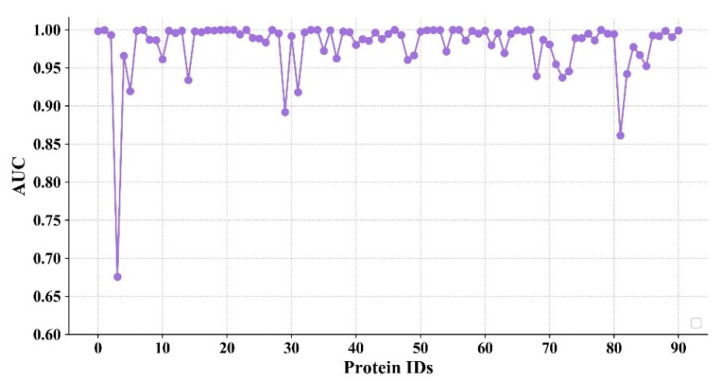
The performance of the model on different proteins.

In order to further evaluate the model’s ability to predict the interaction between different proteins and molecules, in this section, the AUC values for different proteins are shown in Figure 6. The proteins are sorted in the order of Table 2 and Table 3. Since the names of the 91 proteins are long, the corresponding indexes are listed. A total of 23,866 sample pairs are randomly selected for the test (262 sample pairs for each protein on average), which contained 3987 actives and 19,879 decoys.

It can be seen from Figure 6 that the AUC values of most proteins are higher than 0.9, and some of them reach 1. In addition, the number of proteins whose AUC value exceed a predetermined threshold in different models were counted. The comparative results of these models are shown in Table 5. It is worth noting that the number of proteins in the models of AtomNet and Smina is 102. It can be seen from Table 5 that Smina has the lowest prediction accuracy, with 53 proteins exceeding 0.7 and only 1 exceeding 0.9. The AtomNet model is better than Smina, in which the AUC values of 99 targets are higher than 0.7 and the AUC values of 59 targets are over 0.9. However, the AUC values of 88 targets in the proposed model exceed 0.9, which shows that the proposed model is more robust in predicting the compound–protein interaction.

### 4.4. The Analysis of the Model

In order to objectively evaluate the performance of the model and reduce the overfitting to a certain extent, we use 5-fold cross-validation to determine the hyperparameters. When the model was trained well, the average of five results for each hyperparameter combination was regarded as the final result of this set of hyperparameters. Then, the optimal hyperparameter was set to the model, which was retrained on the whole training set and evaluated with the test set.

#### 4.4.1. The Impact of Different Feature Combinations on the Model Performance

In the proposed model for the task of predicting the compound–protein interaction, considering the relevant information of proteins and molecules, the model extracts the local feature (the spatial feature of the binding site) and the global feature (the feature of the composition of the amino acid sequence) from the protein and extracts the topological feature from the molecular structure.

**Figure 7:**
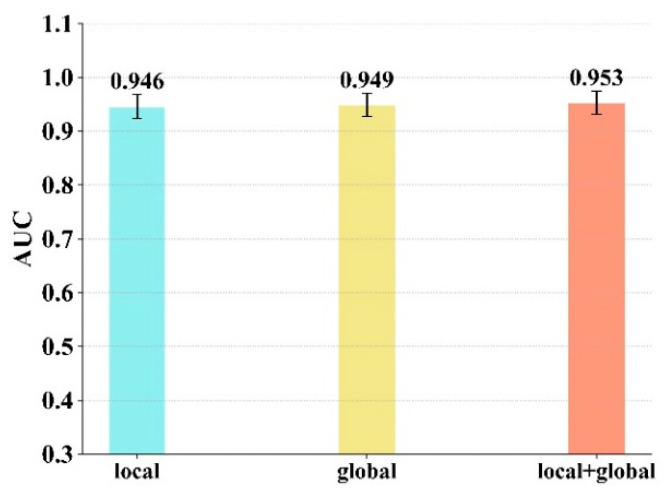
The model performance of 5-fold cross-validation for different feature combinations.

In this section, a comparative experiment was set up to evaluate the impact of different feature combinations, especially different features of the protein. Due to the fact that the molecular feature is indispensable, we combined different features of the protein with the molecular feature. The different feature combinations are: the local feature and molecular feature, the global feature and molecular feature and the combination of all three. The models with different combinations own the same network architecture and hyperparameter configuration. A 5-fold cross validation was performed on all three combinations. From Figure 7, we can see that the combination of the three features achieved the best AUC value.

**Figure 8:**
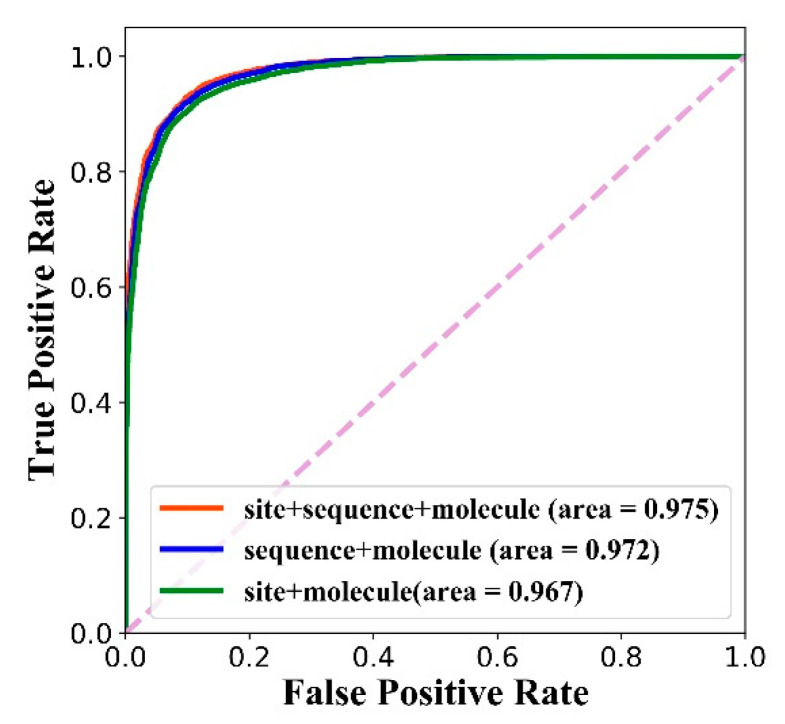
The performance of models with different feature combinations on the test dataset.

After the training is completed, the three models are also tested on the test set, and the ROC curve is shown in Figure 8. We can see that the feature combination of the site structure, amino acid sequence and molecule achieves the best AUC.

Besides, in order to explore the difference between models with three feature combinations, we calculated four indicators: the AUC value, precision value, recall value and $F_{1}$-score. The results are shown in Table 6. The combination of three features achieves the best AUC value, precision value and $F_{1}$-score. It should be noted that the calculation of each precision, recall and $F_{1}$-score is based on specified thresholds. The threshold that achieves the best $F_{1}$-score is determined as the final threshold, which are 0.209, 0.215 and 0.295 for the three feature combinations separately. The results show that the combination of three features (global feature + local feature + molecular feature) is superior to the combination of two features (global feature + molecular feature or local feature + molecular feature).

#### 4.4.2. The Model Performance on Imbalanced Dataset

This section shows the model performance on the balanced and imbalanced dataset. For each ligand, we randomly selected one, three and five decoys consisting of the training dataset, and the ratio of positive and negative is 1:1, 1:3 and 1:5, respectively. Four indicators are set for these three ratios, which includes the AUC value, precision value, recall value and $F_{1}$-score. It can be seen from Table 7 that the AUC value, precision value and $F_{1}$-score of the ratio 1:5 have a little advantage when compared with other ratios. However, the ratio 1:5 is the most imbalanced training dataset. The results show that the imbalance of the dataset has no obvious effect on the model performance.

#### 4.4.3. The Impact of Different Convolutional Layers on the Model Performance

**Figure 9:**
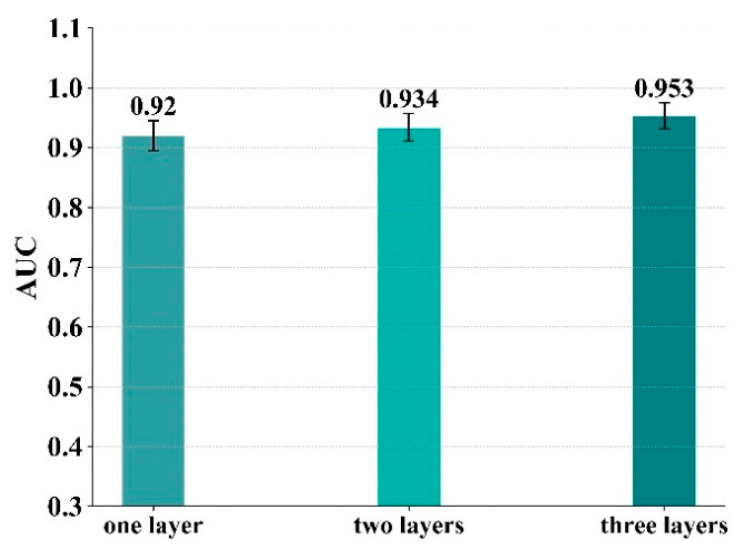
The model performance of 5-fold cross-validation for different convolutional layers.

In the proposed compound–protein interaction predictive model, there are three convolutional networks, including the 3D convolutional network performed on the 3D structure of the binding site, the 1D convolutional network performed on the 1D amino acid sequence of the protein, and the graph convolutional network performed on the molecular structure. Each convolutional network could consist of multiple layers. The purpose of the experiments in this section is to evaluate the influence of different numbers of convolutional layers on the model. In this section, the number of convolutional layers in each model is set from one to three. The number of convolutional layers here corresponds to three convolutional networks (3D convolutional network, 1D convolutional network and graph convolutional network). For example, in a model where the number of convolutional layers is set to one, all three convolutional networks consist of one layer. The results are shown in Figure 9, from which it can be concluded that when the number of convolutional layers is three, the model predicts compound–protein interactions most accurately.

#### 4.4.4. The Impact of Different 3D Convolutional Channels on Model Performance

As for the feature extraction from the spatial structure of the binding site, a multichannel 3D convolutional neural network is applied. The channel setting detail of the proposed model is listed in the first line of Table 8. In this section, in order to evaluate the influence of the number of channels on the performance of the model, two comparative groups are set up. The comparative group is also based on a three-layer 3D convolutional neural network. The difference is the number of channels in each group. The configuration of the different comparative groups is shown in the second and third line in Table 8. Except for the different number of channels of the 3D convolutional neural network, the settings of other hyperparameters are consistent with the proposed model. The validation result is shown in Table 8, from which it can be seen that the model with the higher number of channels (first line in Table 8) achieves the best result.

## 5. Conclusions

In this work, we proposed a compound–protein interaction predictive model based on multiscale convolutional neural networks, which combined the global and local feature of the protein and the feature of molecular topology. Aimed at the binding characteristics of proteins and molecules, three convolutional networks were designed to extract the spatial feature of the binding sites, the feature of amino acid sequences and the feature of molecules. These three features were fed into the model to identify whether the protein and molecules can effectively bind. The model in our work does not rely on the binding conformation of the protein and the molecule, and the experimental results show that the model reaches an AUC value of 0.975, which is better than current deep learning models.

## Figures and Tables

**Table 1:** The attributes of each atom.

No	Attributes	Value	Dimension
1	Atom type	C, N, O, S, F, Si, P, Cl, Br, Mg, Na, Ca, Fe, As, Al, I, B, V, K, Tl, Yb, Sb, Sn, Ag, Pd, Co, Se, Ti, Zn, H, Li, Ge, Cu, Au, Ni, Cd, In, Mn, Zr, Cr, Pt, Hg, Pb, X	44
2	The degree of an atom	0,1,2,3,4,5	6
3	The number of Hs on the atom	0,1,2,3,4,5,6,7,8,9,10	11
4	The number of implicit Hs on the atom	0,1,2,3,4,5,6,7,8,9,10	11
5	Aromatic	1	1
	Total		73

**Table 2:** The target details of the training dataset in DUD-E and the number of positive samples (P) for each target.

ID	P	ID	P	ID	P	ID	P	ID	P
adrb1	247	adrb2	231	akt2	117	Ampc	48	andr	269
aofb	122	bace1	283	Braf	152	cah2	492	cdk2	474
cp2c9	120	csf1r	166	cxcr4	40	Def	102	dhi1	330
drd3	480	dyr	231	esr1	383	esr2	367	fa7	114
fabp4	47	fak1	100	fkb1a	111	fpps	85	gcr	258
glcm	54	hdac2	185	Hivint	100	hivpr	536	hivrt	338
hmdh	170	hs90a	88	hxk4	92	igf1r	148	inha	43
ital	138	jak2	107	kif11	116	Lck	420	mapk2	101
mcr	94	mk01	79	mk10	104	mk14	578	mmp13	572
nos1	100	nram	98	pde5a	398	pgh2	435	plk1	107
pnph	103	ppara	373	Ppard	240	pparg	484	prgr	293
pur2	50	reni	104	rock1	100	rxra	131	sahh	63
src	524	thb	103	try1	449	tryb1	148	tysy	109
urok	162	vgfr2	409	Xiap	100				

**Table 3:** The target details of the test dataset in DUD-E and the number of positive samples (P) for each target.

ID	P	ID	P	ID	P	ID	P	ID	P
aa2ar	482	abl1	182	Aces	453	ada	93	casp3	199
cp3a4	170	egfr	542	fa10	537	fgfr1	139	fnta	592
grik1	101	hdac8	170	Kit	166	kith	57	kpcb	135
pa2ga	99	parp1	508	pgh1	195	ptn1	130	pygm	77
pyrd	111	tgfr1	133	wee1	102				

**Table 4:** The performance of different models on the DUD-E dataset.

Model	Smina [20]	AutoDock Vina [21]	AtomNet [20]	3D-CNN [21]	L+LP+R [32]	Tsubaki’s Model [11]	Lim’s Model [30]	Our Model
AUC	0.696	0.716	0.895	0.868	0.93	0.94	0.968	0.975

**Table 5:** The performance on different proteins.

Model	>0.7	>0.8	>0.9
AtomNet	99	88	59
Smina	53	17	1
Our model	90	90	88

**Table 6:** The comparison of different feature combinations.

Combinations	AUC	Precision	Recall	$F_{1}$-Score
Site + molecule	0.967	0.902	0.918	0.910
Sequence + molecule	0.972	0.890	0.936	0.912
Site + sequence + molecule	0.975	0.905	0.929	0.917

**Table 7:** The model performance on the balanced and imbalanced dataset.

Positive:Negative	AUC	Precision	Recall	$F_{1}$-Score
1:1	0.975	0.905	0.929	0.917
1:3	0.975	0.901	0.938	0.919
1:5	0.977	0.915	0.929	0.922

**Table 8:** The effect of the 3D convolutional channels on the model performance.

Layer1 (in, out)	Layer2 (in, out)	Layer3 (in, out)	AUC (std)
3DCNN (8,32)	3DCNN (32,64)	3DCNN (64,128)	0.953 (0.022)
3DCNN (8,8)	3DCNN (8,4)	3DCNN (4,2)	0.948 (0.022)
3DCNN (8,32)	3DCNN (32,32)	3DCNN (32,8)	0.947 (0.023)

## Data Availability

The data presented in this study are available at https://github.com/ShuangWangCN/MCN_model (accessed on 10 July 2021).
